# Rational design and performance prediction of organic photosensitizer based on TATA^+^ dye for hydrogen production by photocatalytic decomposition of water

**DOI:** 10.3389/fchem.2023.1210501

**Published:** 2023-12-15

**Authors:** Yuening Yu, Zhenqing Yang, Yuhong Xia, Yuzhuo Lv, Wansong Zhang, Chundan Lin, Changjin Shao

**Affiliations:** Beijing Key Laboratory of Optical Detection Technology for Oil and Gas and College of Science, China University of Petroleum, Beijing, China

**Keywords:** photocatalytic hydrogen production, organic photosensitizer, DFT/TDDFT, redox potential, absorption spectrum

## Abstract

In comparison to metal complexes, organic photosensitive dyes employed in photocatalytic hydrogen production exhibit promising developmental prospects. Utilizing the organic dye molecule TA+0 as the foundational structure, a series of innovative organic dyes, denoted as TA1-1 to TA2-6, were systematically designed. Employing first-principles calculations, we methodically explored the modifying effects of diverse electron-donating groups on the R1 and R2 positions to assess their application potential. Our findings reveal that, relative to the experimentally synthesized TATA+03, the TA2-6 molecule boasts a spatial structure conducive to intramolecular electron transfer, showcasing the most negative reduction potential (E_red_ = −2.11 eV) and the maximum reaction driving force (△G^0^_2_ = −1.26 eV). This configuration enhances its compatibility with the reduction catalyst, thereby facilitating efficient hydrogen evolution. The TA2-6 dye demonstrates outstanding photophysical properties and a robust solar energy capture capacity. Its maximum molar extinction coefficient (ε) stands at 2.616 × 10^4^ M^−1^·cm^−1^, representing a remarkable 292.8% improvement over TATA+03. In conclusion, this research underscores the promising potential of the TA2-6 dye as an innovative organic photosensitizer, positioning it as an efficacious component in homogeneous photocatalytic systems.

## 1 Introduction

Hydrogen energy is a renewable and clean substitute for coal and oil. Direct combustion of hydrogen energy will not cause environmental pollution or produce greenhouse gases (Alvarez and Cervantes, 2011; Karadag et al., 2014; Melián et al., 2016; Cha et al., 2017; Moon et al., 2018). Using visible light to drive the decomposition of water can turn solar energy into hydrogen energy, which has great potential to solve the growing global energy crisis and environmental problems (Kerr, 2005; Wang M. et al., 2009; Han and Eisenberg, 2014; Zhang et al., 2015; Yuan et al., 2017). The homogeneous photocatalytic system for hydrogen production from water decomposition is composed of a photosensitizer, catalyst, and sacrificial electron donor, and has low construction cost and better industrial production prospects (Rau et al., 2007; Ozawa and Sakai, 2011; Frischmann et al., 2013; Halpin et al., 2013). Due to the structural variability of the multi-component catalytic hydrogen production system, the activity and stability of the system can be optimized by adjusting its composition. Sufficient light absorption and efficient electron transfer are important factors for the establishment of a high-performance photocatalytic water decomposition hydrogen production process (Wang X. et al., 2009; Artero et al., 2011; Wang et al., 2011; Duf and Eisen Be Rg, 2012; Eckenhoff and Eisenberg, 2012; Han and Eisenberg, 2014). Photosensitizers play an important role in the photocatalytic hydrogen production system by collecting light, generating excited electrons, and promoting intermolecular charge transfer (Rangan et al., 2009; Deponti and Natali, 2016; Yuan et al., 2017).

At present, the dominant photosensitizer is the photosensitizer containing rare metal elements such as Ru, Ir, Pt, Rh, and noble metal elements (Cline et al., 2008; Metz and Bernhard, 2010; Khnayzer et al., 2013; Stoll et al., 2014). The long-term development of hydrogen production from photolysis water has been greatly limited by factors such as their high cost, difficulty in acquisition, and instability in solution (Khnayzer et al., 2013; Stoll et al., 2014). Compared with precious metal complexes, organic dyes have abundant raw materials and low synthesis cost, and have been successfully used in homogeneous photocatalytic hydrogen production systems in the past decade, which has a better development prospect. Examples include fluorescein (Han et al., 2012; Das et al., 2015), Eosin Y(Lazarides et al., 2009; Orain et al., 2014), and rhodamine (McCormick et al., 2010) dyes in the xanthanthraquinone group, acridine (Kotani et al., 2006; Kotani et al., 2007; Fukuzumi, 2008; Gong et al., 2011) and proflavine (Krasna, 1979; 1980) in the azacyclic group, and BODIPY dyes (Sabatini et al., 2011; Durá et al., 2015; Sabatini et al., 2016). Here, Gueret et al. reported that in a homogeneous photocatalytic system consisting of triazatriangulenium (TATA^+^) photosensitive dye, [Co^Ⅲ^(CR14)Cl_2_]^+^ catalyst, and ascorbic acid (HA) electron donor, the intermolecular electron transfer mechanism follows the principle of photosensitizer reduction quenching (Gueret et al., 2018). Shao et al. designed a series of TATA^+^ organic molecules with different side chains based on the TATA^+^ derivative parent (Shao et al., 2020a). Leung et al. conducted a comparison of the turnover numbers (TON) of H_2_ for photosensitizers in the realm of photocatalytic hydrogen production. Their findings indicate the reduced form of TATA^+^ dye in acidic solution stability and hydrogen production performance is much higher than the benchmark noble metal photosensitizer [Ru(bpy)_3_]^2+^ reduced form, which is currently the most active organic photosensitizer in the photocatalytic hydrogen production system (Leung and Lau, 2021). The exceptional visible light photocatalytic hydrogen production performance can be attributed to the flat structure of TATA^+^, the presence of three electron-donating nitrogen atoms, and the promotion of free radical delocalization, all of which enhance the stability of TATA^+^ dyes and prevent their degradation during the photocatalytic process.

The absorption of light energy by photosensitizers serves as a crucial energy source for the photocatalytic hydrogen production reaction, effectively initiating the reaction. Given that the absorption range is concentrated in the visible light region, enhancing the molar extinction coefficient of the dye can significantly augment its visible light absorption capacity, thereby enhancing the overall activity of the hydrogen production system. In the reduction quenching of the excited state photosensitizer, the evolution of the Co catalyst reduced by the reduced state photosensitizer H^+^ to generate H_2_ is the second step of reduction quenching, and also the step of H_2_ generation in the hydrogen production reaction. The reaction free energy at this stage, denoted as △G_2_^0^, directly influences the efficiency of H_2_ evolution. The negative reduction potential plays a crucial role in reducing the H_2_ evolution catalyst during the photocatalytic hydrogen production experiment, facilitating the reduced form of the photosensitizer to transfer electrons to the catalyst, thereby enhancing hydrogen production performance. Consequently, reducing the reduction potential of the photosensitizer holds great significance in promoting the photosensitizer reduction of the Co catalyst.

**FIGURE 1:**
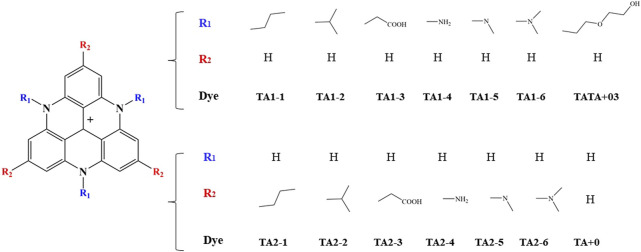
Molecular structures of TA1-1 to TA2-6 simplified model.

Organic photosensitizers offer greater molecular-level tunability compared to precious metal complexes (Han and Eisenberg, 2014). The introduction of suitable functional groups based on the structure-activity relationship enables the adjustment of their redox properties and light absorption capacity (Li et al., 2018). As shown in Figure 1, we have designed a series of organic dyes without metal elements based on TATA^+^ dyes and explored their potential ability to be used as light-driven hydrogen production materials relative to the experimental parent.

## 2 Computational details

Considering the computational cost, we designed a series of new organic dye molecules by screening some functional groups according to the induction effect and conjugation effect of the group itself. In Figure 1, TA1-1 to TA-6 denote n-propyl, isopropyl, methyl carboxyl, amino, methylamino, and dimethylamino, respectively. Functional groups were introduced into the nitrogen position (R1 position) and R2 position of the TA+0 molecule, respectively, to optimize the ground state structure of the molecule, reduce its reduction potential, and improve its photophysical properties. It is of positive significance to the efficiency of capturing sunlight as a photosensitizer for hydrogen evolution systems and the ability of reducing the Co catalyst in the reaction.

All the calculations in this paper are carried out in the Gaussian 09 software package (Patterson, 1989; Frisch et al., 2009). Different exchange correlation functions (XC) usually have a significant effect on charge transfer excitation. Compared with the experimental results, these methods are considered to be reliable (Shao et al., 2020b). Based on density functional theory (DFT) and HCTH function combined with the 6-311+G (d, p) basis group, the ground state geometry of dye molecules was optimized in the conductor polarized continuum model (CPCM) in acetonitrile solvent (Frisch et al., 1984; Patterson, 1989; Hamprecht et al., 1998). Using Gauss View 5.0.8, the HOMO-LUMO front molecular orbital electron density and energy level analyses were performed on the ground state optimization results. The excited states were calculated in the conductor polarized continuum model (CPCM) of acetonitrile solvent by using the time-dependent density functional theory (TD-DFT) and the 6-311+G (d, p) basis group combined with the HCTH function to predict the photophysical properties of dyes (Frisch et al., 1984; Bauernschmitt and Ahlrichs, 1996; Hamprecht et al., 1998).

## 3 Results and discussion

### 3.1 Electrochemical and photophysical properties

Using the spatial characteristics of electron transfer to regulate the redox potential of photosensitive dyes is beneficial to improve the photocatalytic performance and build efficient hydrogen production systems. After ground state structure optimization using DFT, HOMO and LUMO levels of dye molecules can be obtained, as shown in Table 1. Formulas (1) and (2) reflect the electrochemical relationship between energy level and redox potential:

$$E_{LUMO}=-4.5\text{eV}-\left(E_{red}+0.24\right)$$ (1)

$$E_{HOMO}=-4.5\text{eV}-\left(E_{ox}+0.24\right)$$ (2)

where E_red_ and E_ox_ are the reduction potential and oxidation potential of the dye, respectively, representing the relationship with the saturated calomel electrode (SCE). The more negative reduction potential is favorable for the photosensitizer to transfer electrons to the catalyst. In all the designed molecules except for the carboxyl methyl group substituted TA1-3 and TA2-3, the absolute value of the reduction potential was improved to some extent, and the reducing ability of the dye was enhanced. The reduction potential of the excited state of the dye can be obtained from formula (3) (Prier et al., 2013; Yuan et al., 2017):

$$E_{red}^{*}=E_{red}+E_{0-0}$$ (3)

where E_0-0_ represents the vertical excitation energy of the dye calculated by TD-DFT method. The redox potentials of all dyes are shown in Table 1.

**TABLE 1:** Calculated electrochemical data for dyes: highest occupied molecular orbital energy level (E_HOMO_), lowest unoccupied molecular orbital level (E_LUMO_), excited state energy (E_0-0_), reduction potential (E_red_), first oxidation potential (E_ox_), and excited state reduction potential (E^*^_red_) by HCTH/6-311G+(d, p) and TD-HCTH/6-311G+ (d, p).

Dyes	HOMO/eV	LUMO/eV	E_0-0_/eV	E_red_/V	E_ox_/V	E^*^_red_/V
TA+0	−5.54	−3.43	2.39	−1.31	0.80	1.08
TATA+03	−5.58	−3.52	2.34	−1.22	0.84	1.12
TA1-1	−5.47	−3.38	2.36	−1.36	0.73	1.01
TA2-1	−6.00	−2.71	2.41	−2.03	1.26	0.38
TA1-2	−5.42	−3.45	2.25	−1.29	0.68	0.97
TA2-2	−5.84	−2.94	2.44	−1.80	1.10	0.64
TA1-3	−5.75	−3.68	2.36	−1.06	1.01	1.30
TA2-3	−5.63	−3.52	2.37	−1.22	0.89	1.16
TA1-4	−5.58	−3.50	2.37	−1.24	0.84	1.13
TA2-4	−5.26	−2.76	3.08	−1.98	0.52	1.10
TA1-5	−5.44	−3.45	2.30	−1.29	0.70	1.01
TA2-5	−5.09	−2.64	2.74	−2.10	0.35	0.64
TA1-6	−5.51	−3.48	2.36	−1.26	0.77	1.10
TA2-6	−5.04	−2.63	2.68	−2.11	0.30	0.57

Only when the reduction potential of the excited dye is higher than the oxidation potential of the electron donor can the excited photosensitizer be reduced and quenched by the electron donor. In this hydrogen production system, the oxidation potential of ascorbic acid as the sacrificial electron donor is E_HA•/HA-_ = 0.11 V (vs. SCE) (Eckenhoff and Eisenberg, 2012; Gueret et al., 2018). For the second step of reduction quenching, the reduction potential E_red_ of the dye should be negative to the reduction potential of the Co catalyst, where E_Co_^Ⅱ^_/Co_^Ι^ = −0.85 V (vs. SCE) (vs. SCE) takes the experimental value of Co^Ⅱ^/Co^Ι^ in an aqueous solution (Gueret et al., 2018). As shown in Table 1, the redox potentials of all dyes meet the level matching condition in reduction quenching.

According to the Rehm–Weller equation, the reduction driving forces △G_1_^0^ and △G_2_^0^ of the reduction quenching of the excited photosensitizer are given by formula (4) and (5) (Rehm and Weller, 1970; Lomoth and Ott, 2009; Wang et al., 2012):

$${∆G}_{1}^{0}\left(\text{eV}\right)=E_{{HA}^{\cdot }/{HA}^{-}}-E_{PS/{PS}^{-}}-E_{0-0}-C$$ (4)

$${∆G}_{2}^{0}\left(\text{eV}\right)=E_{PS/{PS}^{-}}-E_{{Co}^{Ⅱ}/{Co}^{Ι}}$$ (5)

where C represents the sum of the solvation effect and Coulomb energy of the ion pair in solution, which is ignored as 0.

As shown in Table 2, the reaction driving forces △G_1_^0^ and △G_2_^0^ of all dyes are less than 0, which indicates that within the range of thermodynamic tolerance, electron transfer can be carried out spontaneously from the sacrificed-electron donor to the excited state photosensitizer and from the reduced-form photosensitizer to the Co catalyst to complete the reduced-quenching photocatalytic hydrogen production reaction. The more negative the reduction driving force △G_2_^0^ shows that the stronger the reduction capacity of the Co catalyst, the more favorable the charge transfer from photosensitizer to catalyst. By proper modification of the R1 and R2 sites of the molecule, more negative E_red_ and △G_2_^0^ can be obtained, which helps the photosensitizer to reduce the catalyst to further have a positive significance for hydrogen generation.

**TABLE 2:** Calculated maximum absorption wavelengths (λ_max_), maximum molar extinction coefficient (ε), oscillator strength (f), and injection driving force (ΔG^0^_1_, and ΔG^0^_2_) of all the dyes.

Dyes	λ_max_/nm	ε/(10^3^·M^−1^·cm^−1^)	f	△G_1_^0^/eV	△G_2_^0^/eV
TA+0	519.6	3.56	0.0439	−0.97	−0.46
TATA+03	530.00	6.66	0.0822	−1.01	−0.37
TA1-1	525.20	6.82	0.0843	−0.90	−0.51
TA2-1	514.00	3.74	0.0463	−0.27	−1.18
TA1-2	550.40	6.26	0.0773	−0.86	−0.44
TA2-2	508.00	3.66	0.0470	−0.53	−0.95
TA1-3	526.00	7.00	0.0867	−1.19	−0.21
TA2-3	522.40	3.72	0.0472	−1.05	−0.37
TA1-4	522.40	5.10	0.0629	−1.02	−0.39
TA2-4	410.00	16.57	0.1611	−0.99	−1.13
TA1-5	543.00	6.43	0.0838	−0.90	−0.44
TA2-5	437.40	22.79	0.1931	−0.53	−1.25
TA1-6	529.60	6.37	0.0793	−0.99	−0.41
TA2-6	460.80	26.16	0.2844	−0.46	−1.26

**FIGURE 2:**
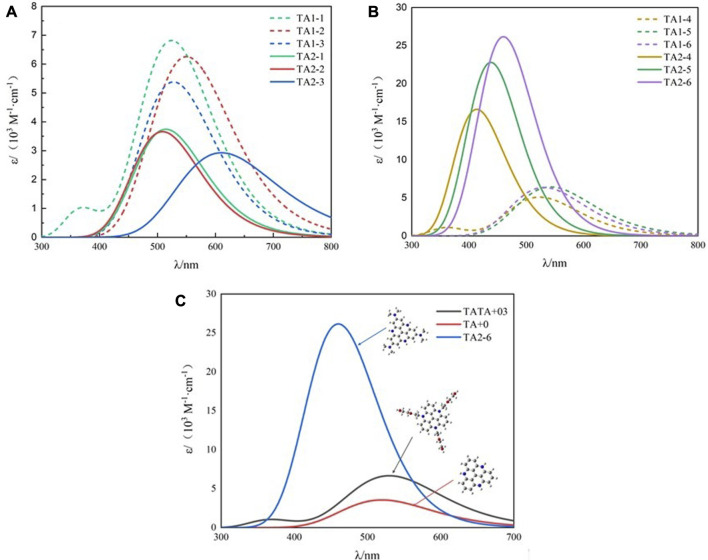
Calculated UV−visible absorption spectra by TD-HCTH/6-311+G (d, p) in acetonitrile **(A)** TA1-1∼TA2-3, **(B)** TA1-4∼TA2-6, **(C)** TA+03, TA+0, and TA2-6.

As the main body of light collection, the photosensitizer can improve the utilization rate of light energy and contribute to the design of low-cost and efficient hydrogen production reactions. Figure 2 shows the absorption spectra of all the dyes calculated in acetonitrile solution. The light capture ability and excited photophysical properties of dye molecules are regulated by modifying their structure. Table 2 shows the photophysical data we calculated using TD-DFT, including the maximum molar extinction coefficient and the position of the absorption peak. Compared with the parent structure TA+0, the maximum molar extinction coefficients of the dye molecules modified by weak electron-donating groups at R1 and strong electron-donating groups at R2 were significantly improved.

### 3.2 The role of weak electron-donating groups

The side chains at the R1 and R2 positions of dyes will have a strong effect on their hydrogen production performance in visible-light-driven hydrogen production (Shao et al., 2020b; Lin et al., 2021). When the functional groups were modified at different positions of TA+0 matrix, the molecular orbital energy level, light absorption capacity, and reduction potential level of the dye were significantly affected.

TA1-1, TA1-2, and TA1-3 are obtained when the weak electron-donating group is connected to the R1 position of TA+0 and the R2 position retains the H atom. Their maximum molar extinction coefficients were 6.82×10^3^·M^−1^·cm^−1^, 6.26×10^3^·M^−1^·cm^−1^, and 6.26×10^3^·M^−1^·cm^−1^, which were higher than the 3.56×10^3^·M^−1^·cm^−1^ of the parent molecule TA+0. When the R2 position is connected to the substituents and the R1 position retains H atoms, the reduction potential E_red_ and the reduction driving force △G_2_^0^ of the TA2-1 and TA2-2 dyes become more negative, indicating that these dyes have stronger reduction ability and are more favorable to the activation of catalysts. However, the energy level and free energy of TA2-3 dye do not change to a more negative direction, because the methyl carboxyl group has weak electron-donating ability and does not show a good electron-donating effect after connecting with the TA+0 matrix. Nevertheless, they are not suitable for photocatalytic hydrogen production system, because the molar extinction coefficient of these three dyes is too small to absorb visible light well. We found that the modification of R1 position by the weak electron-donating group can effectively improve the photophysical properties of dyes based on the TA+0 matrix.

### 3.3 The role of strong electron-donating groups

However, on further investigation, we found something even more interesting. When we attached more electron-donating groups at R1 and R2, substituting R1 and R2 had different effects on the parent molecule than substituting weak electron-donating groups. For the functional groups added by the latter six dye molecules, the relationship between the electron-giving ability is as follows: NCH_3_CH_3_ (dimethyl amino) > -NHCH_3_ (methyl amino) > -NH_2_ (amino).

When they are connected at R1 position, they have no obvious effect on the negative change of the orbital energy level and reduction potential of the photosensitizer. This may be due to the orbital overlap and interaction between the nitrogen atoms on these three groups and the nitrogen atoms at the R1 replacement position, resulting in the failure of the three strong electron-donating groups to show outstanding electron-donating effects. Compared with TATA+03, the absolute value of TA1-4, TA1-5, and TA1-6 reduction potentials only increased by 0.02 eV, 0.07 eV, and 0.04 eV, respectively. Compared with the parent molecule TA+0, its molar extinction coefficient also increased, but the calculated value of the same level did not exceed that of the experimental synthesis TATA+03 dye.

**FIGURE 3:**
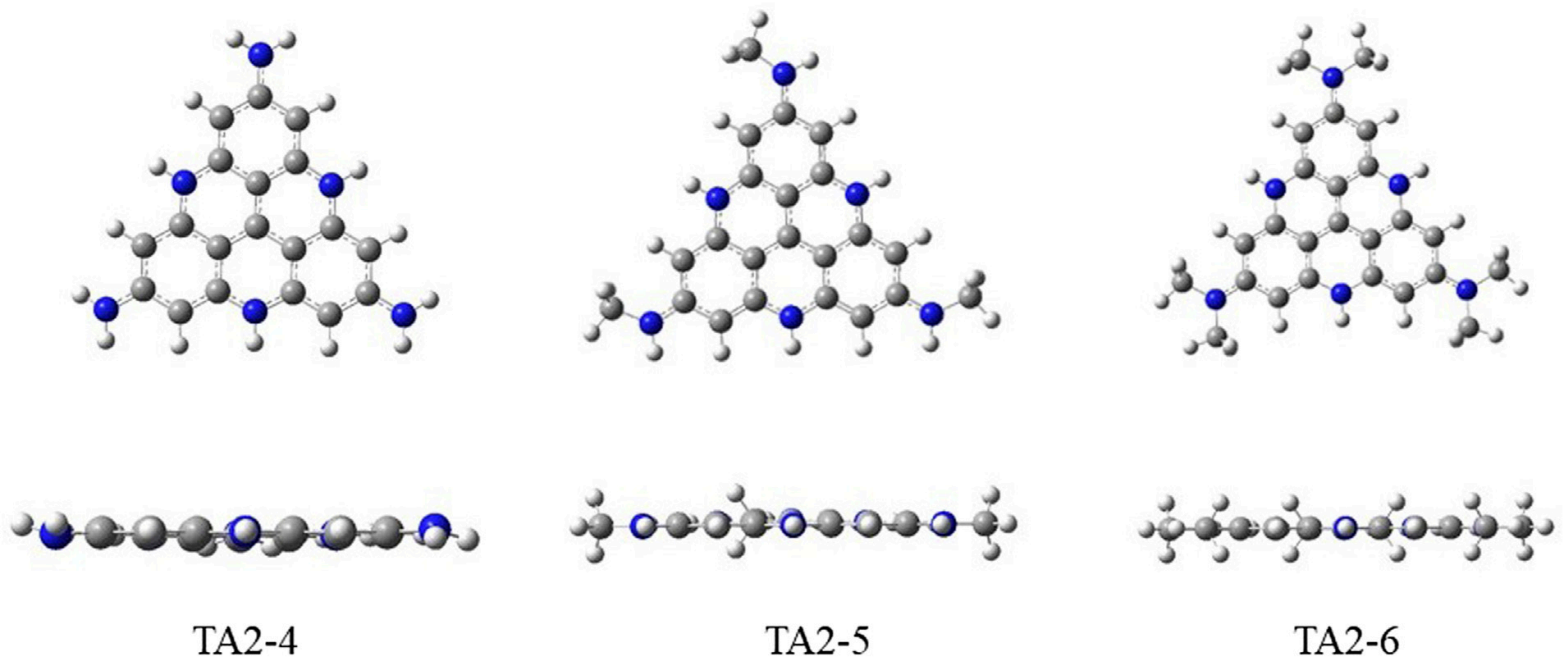
The optimized structures of TA2-4, TA2-5 and TA2-6 by HCTH/6-311G+ (d, p).

When they were connected to the carbon atom in R2 position, TA2-4, TA2-5, and TA2-6 showed the most negative reduction potential and the strongest reaction driving force. Compared with TATA+03, they respectively had a larger negative reduction potential of −1.98 eV, −2.10 eV, and −2.11 eV, and a more negative reduction potential of 62.3%, 72.1%, and 73%. The △G_2_^0^ of the three dyes reached −1.13 eV, −1.25 eV, and −1.26 eV, respectively, showing a stronger ability of reducing the Co catalyst. This is because the structure modification of the strong electron-donating group increases the density of the electron cloud on the parent molecule, showing a strong electron-donating effect. As shown in Figure 3, their geometries optimized in the ground state are studied. It can be seen that the functional group and the benzene ring structure of the parent molecule show excellent flatness. The nitrogen atoms of the substituent group and the benzene ring plane of the parent molecule are located in the same plane, which is more conducive to promoting the charge transfer inside the molecule.

**FIGURE 4:**
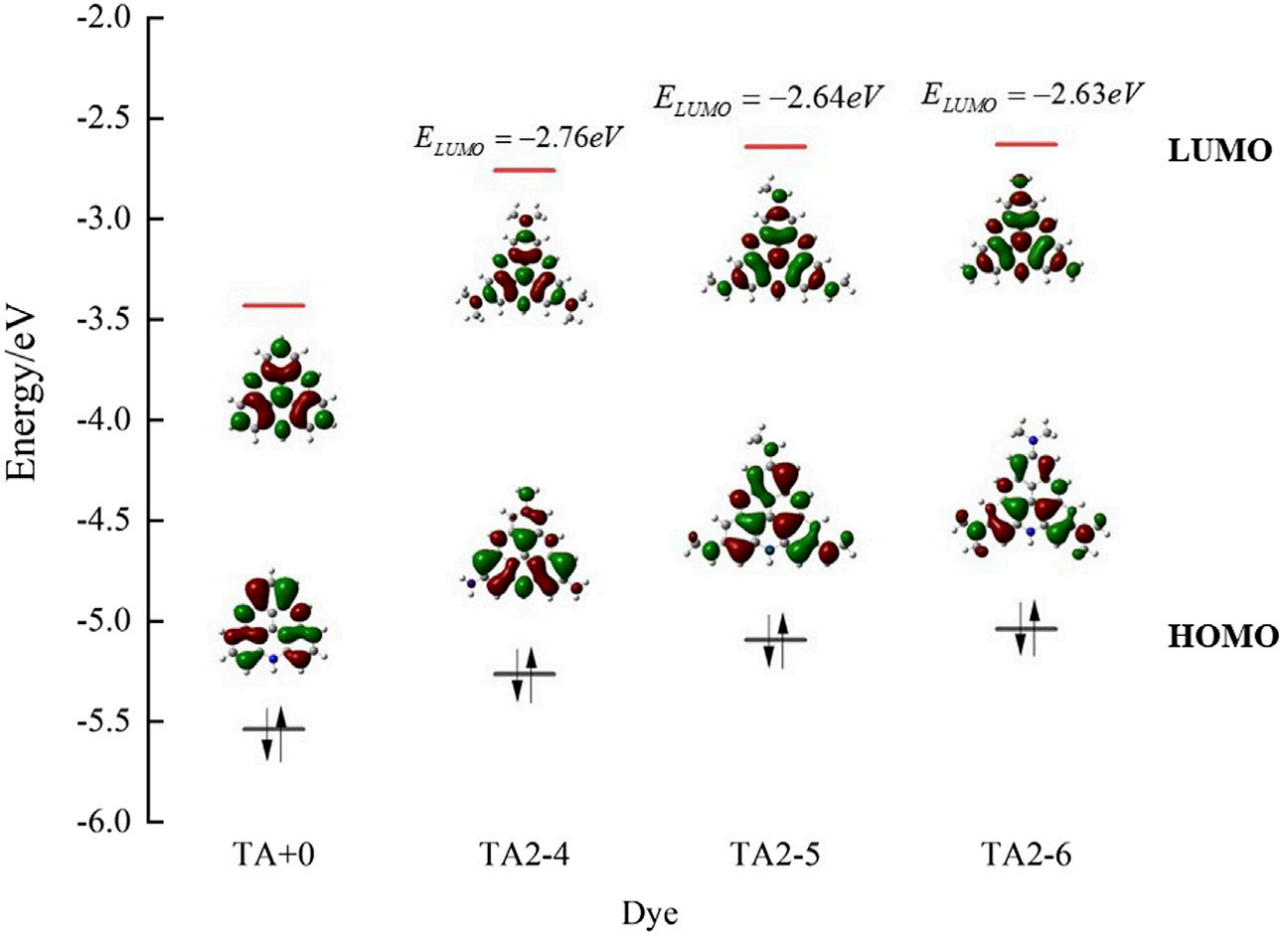
Isodensity plots for the HOMO and LUMO levels and calculated energy levels of TA+0, TA2-4, TA2-5 and TA2-6.

As shown in Figure 3, we found that TA2-4, TA2-5, and TA2-6 exhibit excellent absorption of light energy because these substituents are also chromophores. The molar extinction coefficient of TATA+03 is 6.66 × 10^3^·M^−1^·cm^−1^. The molar extinction coefficients of dyes TA2-4, TA2-5, and TA2-6 were 1.657 × 10^4^·M^−1^·cm^−1^, 2.279 × 10^4^·M^−1^·cm^−1^, and 2.616 × 10^4^·M^−1^·cm^−1^, respectively, with increments of 148.8%, 242.2%, and 292.8%. The high molar extinction coefficient shows the excellent light capture ability of dyes, which can effectively absorb solar energy and improve the utilization rate of light energy for movable hydrogen reaction of optical drive and photocatalytic performance. Figure 4 shows the isodensity plots for the HOMO and LUMO levels and calculated energy levels of TA+0, TA2-4, TA2-5 and TA2-6. Although their absorption peak positions have a certain blue shift phenomenon, this is because the increase of the LUMO energy level of the dye makes the dye have more negative reduction potential, which also increases the band gap of the molecule, resulting in the required increase of the electron transition.

In contrast, the absorption peak position of TA2-6 dye is 460 nm, and the absorption range covers more visible regions and has the highest molar extinction coefficient, the most negative reduction potential, and reduction driving force. Considering that △G_1_^0^ = −0.46 eV, although it is not good for the electron donor reduction of an excited state photosensitizer, the reduced dye is still very favorable for the activation of a catalyst, which is favorable for H_2_ release. Therefore, TA2-6 is considered to be the most promising organic photosensitive dye, which can be combined with ascorbic acid and [Co^Ⅲ^(CR14)Cl_2_]^+^ to construct efficient photocatalytic hydrogen production systems.

## 4 Conclusion

In this study, leveraging the organic dye molecule TA+0 as the foundational matrix, we conducted a systematic exploration of R1 and R2 positions through the introduction of electron-donating groups with varying intensities. Subsequently, a series of meticulously designed dye molecules, denoted as TA1-1 to TA2-6, were synthesized. Employing first principles, we meticulously investigated the ground state structure, energy gap, reduction potential, reaction driving force, and UV-visible absorption spectrum of these novel dye molecules. The findings elucidate that the augmentation of both the reduction ability and light absorption in the dye was achieved by strategically incorporating three potent electron-donating groups at the R2 position for structural refinement. Notably, in contrast to TATA+03, TA2-6 exhibited a spatial configuration conducive to intramolecular electron transfer. Furthermore, the observed highly negative reduction potential (E_red_ = −2.11 eV) and substantial reaction driving force (△G_2_^0^ = −1.26 eV) in TA2-6 are particularly advantageous for the progression of the reduction catalyst and efficient hydrogen generation. The exceptional photophysical properties of TA2-6, characterized by a molar extinction coefficient of 2.616×10^4^·M^−1^·cm^−1^, represent a remarkable 292.8% increase over TATA+03. These compelling results underscore the potential of TA2-6 as a promising organic dye, positioning it as a novel organic photosensitizer for the development of a highly efficient homogeneous photocatalytic system. Beyond these immediate implications, our study is poised to offer crucial theoretical support for the advancement of more efficient homogeneous non-noble metal photocatalytic hydrogen production systems.

## Data Availability

The original contributions presented in the study are included in the article/Supplementary Material, further inquiries can be directed to the corresponding authors.
