# Supplementary material for: Rational design and performance prediction of organic photosensitizer based on TATA+ dye for hydrogen production by photocatalytic decomposition of water
Source: Front Chem. 2023 Dec 15;11:1210501. doi: 10.3389/fchem.2023.1210501 (PMC10757343; doi:10.3389/fchem.2023.1210501)
Supplement: Supplementary file 1 [file DataSheet1.docx]

Supplementary Material

# Supplementary Figures


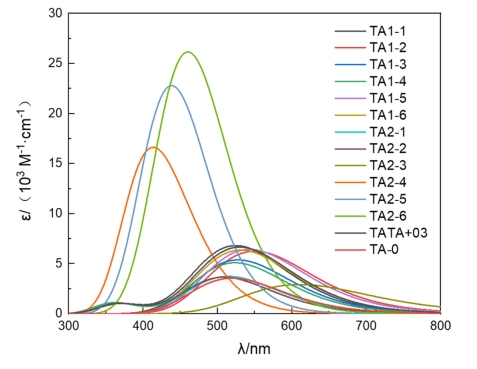


**Supplementary Figure 1.** Calculated absorption spectrum of **TA1-1~TA-0** by HCTH/6-311+ (d, p).


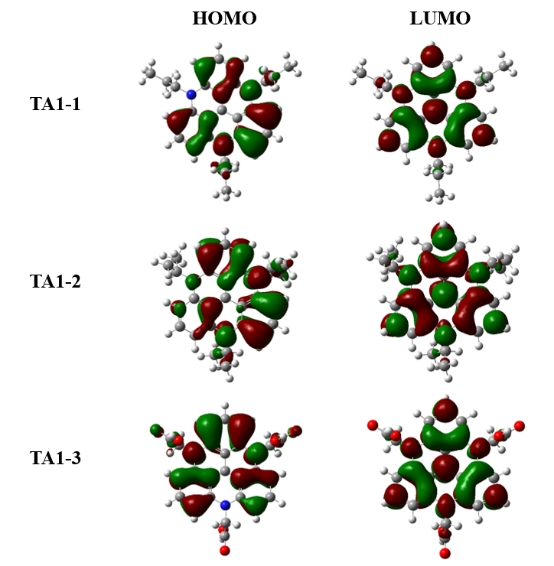


**Supplementary Figure 2.** Frontier molecular orbitals for **TA1-1~TA1-3**.


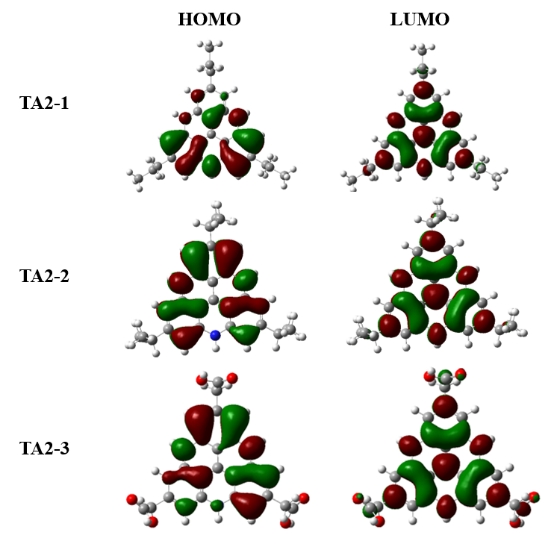


**Supplementary Figure 3.** Frontier molecular orbitals for **TA2-1~TA2-3**.


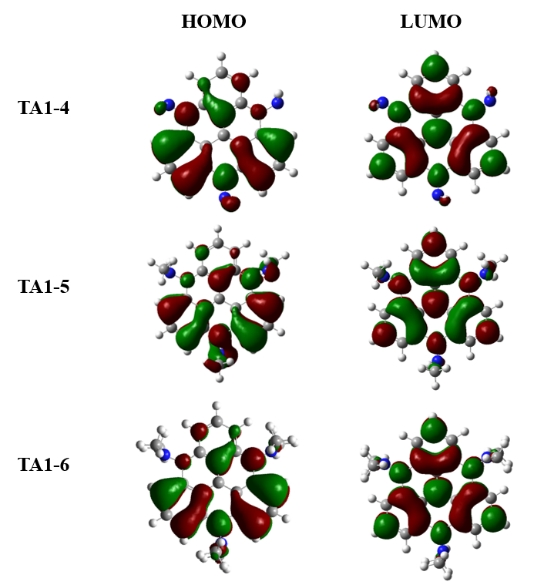


**Supplementary Figure 4.** Frontier molecular orbitals for **TA1-4~TA1-5**.


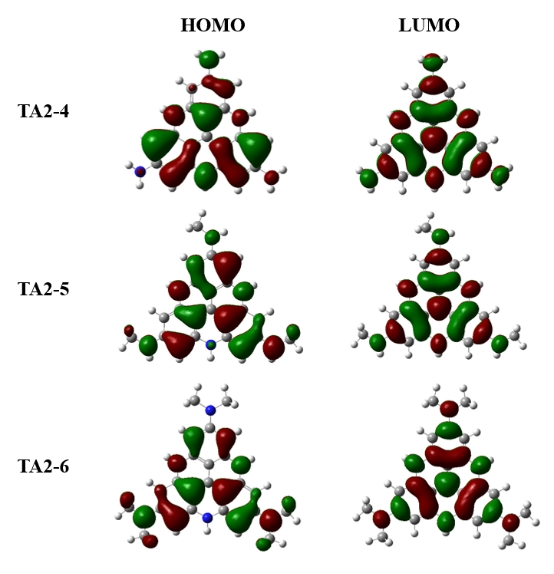


**Supplementary Figure 5.** Frontier molecular orbitals for **TA2-4~TA2-6**.
